# Augmented Backward Elimination: A Pragmatic and Purposeful Way to Develop Statistical Models

**DOI:** 10.1371/journal.pone.0113677

**Published:** 2014-11-21

**Authors:** Daniela Dunkler, Max Plischke, Karen Leffondré, Georg Heinze

**Affiliations:** 1 Medical University of Vienna, Center for Medical Statistics, Informatics and Intelligent Systems, Section for Clinical Biometrics, Vienna, Austria; 2 Medical University of Vienna, Division of Nephrology and Dialysis, Department of Internal Medicine III, Vienna, Austria; 3 Université Bordeaux Segalen, ISPED, Centre de recherche INSERM U897, Bordeaux, France; University of New South Wales, Australia

## Abstract

Statistical models are simple mathematical rules derived from empirical data describing the association between an outcome and several explanatory variables. In a typical modeling situation statistical analysis often involves a large number of potential explanatory variables and frequently only partial subject-matter knowledge is available. Therefore, selecting the most suitable variables for a model in an objective and practical manner is usually a non-trivial task. We briefly revisit the purposeful variable selection procedure suggested by Hosmer and Lemeshow which combines significance and change-in-estimate criteria for variable selection and critically discuss the change-in-estimate criterion. We show that using a significance-based threshold for the change-in-estimate criterion reduces to a simple significance-based selection of variables, as if the change-in-estimate criterion is not considered at all. Various extensions to the purposeful variable selection procedure are suggested. We propose to use backward elimination augmented with a standardized change-in-estimate criterion on the quantity of interest usually reported and interpreted in a model for variable selection. Augmented backward elimination has been implemented in a SAS macro for linear, logistic and Cox proportional hazards regression. The algorithm and its implementation were evaluated by means of a simulation study. Augmented backward elimination tends to select larger models than backward elimination and approximates the unselected model up to negligible differences in point estimates of the regression coefficients. On average, regression coefficients obtained after applying augmented backward elimination were less biased relative to the coefficients of correctly specified models than after backward elimination. In summary, we propose augmented backward elimination as a reproducible variable selection algorithm that gives the analyst more flexibility in adopting model selection to a specific statistical modeling situation.

## Introduction

Statistical modeling is concerned with finding a simple general rule to describe the dependency of an outcome on several explanatory variables. Such rules may be simple linear combinations, or more complex formulas involving product and non-linear terms. Generally, statistical models should fulfill two requirements. First, they should be *valid*, i.e., provide predictions with acceptable accuracy. Second, they should be *practically useful*, i.e., a model should allow to derive conclusions such as ‘how large is the expected change in the outcome if one of the explanatory variables changes by one unit’. In a typical modeling situation the analyst is often confronted with a large number of potential explanatory variables, and selecting the most suitable ones for a model is usually a non-trivial task.

Statistical models are used in predictive as well as in etiologic research [1]. In the former, one is interested in a simple and well-interpretable rule in order to accurately predict an outcome of interest, while in the latter, the strength of an assumed relationship of a variable of interest, i.e., the exposure variable, with an outcome is investigated. Control of confounding by multivariable adjustment (or other techniques such as propensity scores) is crucial if such relationships are to be estimated from observational rather than from randomized intervention studies [2], [3]. Thus, in both types of research valid and useful statistical models are needed.

Backward, forward, and stepwise variable selection algorithms are implemented in most regression software packages, and together with univariate screening they are the algorithms that are used most often to select variables in practice (see e.g. [4], [5] Chapter 2). All these algorithms rely only on significance as a sufficient condition to include variables into a model. For example, univariate screening includes variables based on the significance of their associations with the outcome in univariate models, or backward elimination removes insignificant variables one-by-one from a model. An excellent, critical summary of standard variable selection methods can be found in Royston and Sauerbrei ([5], Chapter 2).

Hosmer and Lemeshow proposed the ‘purposeful selection algorithm’ [6], [7] which combines significance and change-in-estimate criteria [8]–[12] for selecting explanatory variables for a final model and is particularly attractive as it can be realized with standard software. Here, we will readopt the idea of combining significance and change-in-estimate criteria, and we will suggest a simple approximation to quantify the change-in-estimate from which a hypothesis test on the change-in-estimate can be directly derived.

The remainder of the manuscript is organized as follows: the Methods section will first discuss the change-in-estimate criterion and selection by significance. Later, we will present a new proposal for an efficient algorithm, denoted as augmented backward elimination (ABE), combining both criteria. A SAS macro incorporating the ABE algorithm will be introduced [13], [14]. The subsequent section summarizes results of a simulation study to evaluate the algorithm. Aspects of application of ABE are discussed by means of a study of progression of chronic kidney disease, including the use of resampling methods for confidence interval estimation and for assessing model stability.

## Methods

### The Change-In-Estimate Criterion Revisited

We denote by 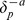 the change-in-estimate, i.e., the change in a regression coefficient 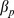 by removal of a variable 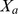 from an arbitrary linear statistical model with 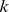 explanatory variables 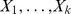; 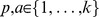; 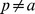. (The indices 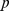 and 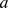 refer to the roles of *X_p_* and *X_a_* in 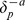 as the ‘*p*assive’ and ‘*a*ctive’ variables, respectively.) Instead of refitting the model with 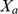 omitted, we propose to approximate the change-in-estimate, using the estimates 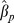 and 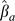, their covariance 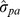, and the variance of 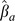, 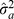, as
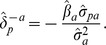



This approximation is motivated by considering 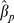 and 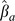 as random variables with variances 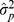 and 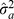 and covariance 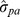. The slope of a regression of 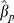 on 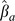, which denotes the expected change in 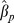 if 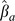 is augmented by 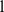, is then given by 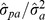. Since we would like to approximate what happens if 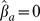, i.e., if 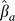 is subtracted from 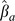, we multiply the slope by 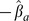. The approximation does not only speed up the evaluation of the change-in-estimate considerably, but it also allows to directly assess the ‘significance’ of the change-in-estimate, i.e., to test for collapsibility of the models including and excluding 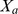. The variance of 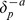 is given by 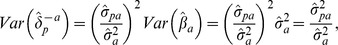
and its standard error follows as 
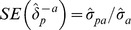
. If the covariance 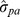 is not exactly zero, a *z*-statistic for testing 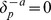 could thus be constructed by
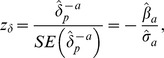
which equals the *z*-statistic for testing 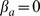. Thus, removal of ‘significant’ active variables causes ‘significant’ changes in the estimates of passive variables, and removal of ‘non-significant’ active variables causes ‘non-significant’ changes in the estimates of passive variables. Consequently, attempting to use a significance-based threshold for the change-in-estimate criterion reduces to a simple significance-based selection of variables, as if the change-in-estimate criterion is not considered at all. (If the covariance 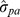 is exactly zero, then 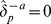 irrespective of 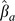, and elimination of 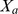 will not cause a change in 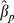. This case can only be expected in analyses of experiments with factorial designs by linear models, a situation where variable selection is not considered.)

Under the null hypothesis of 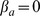, equivalent to 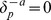, variable selection based on significance-testing will control the probability of falsely selecting 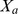 approximately at the nominal type I error rate. However, the change-in-estimate criterion is usually evaluated using a pre-specified minimum value of 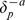 or 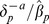 as a threshold for leaving 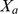 in a model [10], [12], [15], and thus the probability of a false selection of 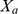 is not controlled. This probability is rather associated with the standard error of 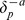, which is higher in smaller samples compared to larger ones.

Despite this unfavorable property, the change-in-estimate criterion may still be useful to obtain a model which approximates the unselected model up to negligible differences in point estimates of the regression coefficients, but contains fewer variables. Another justification for incorporating the change-in-estimate criterion in variable selection is to avoid the tendency of purely significance-based selection to select only one out of several correlated variables.

Some authors used a relative criterion 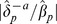 with, e.g., 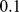 as the threshold value [6], [10], [12], [15]. This definition may not be suitable if 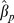 is close to zero. We propose the following criteria, which do not share this property, are suitably standardized and focus on the quantities of interest in a regression analysis:

1. In linear regression, regression coefficients depend on the scaling of the explanatory variable and on that of the outcome variable. Scale-independence is attained by evaluating

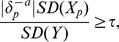
where 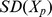 and 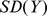 are the standard deviations of the passive explanatory variable 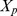 and the outcome 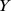, respectively.

1. In logistic or Cox regression, interest lies in odds and hazard ratios, respectively. This leads us to the standardized criterion

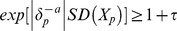
 or equivalently, 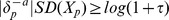.

The threshold value 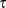 could be set to, say, 0.05 but can be adopted to the specific modeling situation.

Usually, the individual explanatory variables play different roles (e.g., exposure variable of interest, important adjustment variable, less important adjustment variable) and this should be reflected in the selection process. We have identified three specific roles of explanatory variables, which may require different handling when evaluating the change-in-estimate criterion:

1. ‘Passive or active’ explanatory variables: Generally, explanatory variables are used as passive as well as active variables when evaluating the change-in-estimate criterion.
2. ‘Only passive’ explanatory variables: In etiologic modeling, it is necessary to always keep the exposure variable of interest in the model. Furthermore, one may force the modeling process to always include some known confounders (in etiologic modeling) or predictors (in prognostic modeling). Such exposures of interest, known confounders or predictors are always considered as passive variables in evaluating the change-in-estimate criterion for other variables.
3. ‘Only active’ explanatory variables: Less important explanatory variables should only be included if their exclusion causes changes in the estimates of more important explanatory variables. Thus, such variables of minor importance are only considered as active but never as passive variables when evaluating the change-in-estimate criterion.

### Variable Selection Based on Significance

Most variable selection procedures that are used in practice only rely on significance, e.g., univariate screening, forward selection, stepwise selection, and backward elimination (BE). Literature suggests that BE procedures with a mild significance level criterion, e.g., 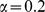, are superior to other approaches with regard to bias and root mean squared error of regression coefficients [8], [9], [16], [17]. BE has a tendency to under-select important confounders [18], because it ignores variables with a strong association with the exposure, but a relatively weak association with the outcome conditional on the exposure. Royston and Sauerbrei also distinguish between ‘BE only’ and BE with additional forward steps, in which variables that have already been excluded at earlier iterations are reconsidered for inclusion [5]. They conclude that re-inclusion after exclusion rarely occurs. Therefore, we consider BE-only with a significance criterion of 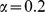 as the consensus method for significance-based variable selection. There is no statistical justification for univariate screening ([17], [19] Chapter 4.4). Forward selection may sometimes be preferred over BE for practical reasons, e.g., in very high-dimensional variable selection problems. Stepwise selection, e.g., as implemented in SAS procedures [13], is essentially a forward selection with additional backward steps.

### The Initial Working Set of Variables

For estimating etiologic models *a priori* information should be used to define the initial working set of variables to consider during statistical modeling. This *a priori* information can often be represented by a directed acyclic graph (DAG) which reflects the conditional dependencies of variables [20], [21]. DAGs prompt the analyst to carefully question the causal relationship between all explanatory variables in a model, and they allow to identify the role of each variable: either as a confounder, a mediator, a variable unrelated to the causal relationship of interest [22], or incorporating the possibility of unmeasured quantities, a collider [23]. Finally, only variables assumed to be confounders, i.e., variables which are possibly associated with the outcome and with the exposure variable of interest, but which are not on the causal pathway from the exposure to the outcome, should be included for multivariable adjustment. Application of such causal diagrams requires that the analyst knows how each explanatory variable is causally related to each other [24]. However, in many areas of research such knowledge is hardly available or at least very uncertain.

For prognostic modeling situations the initial set of variables will be selected based on other reasons, like future availability, the costs of collecting these variables, the reliability of measurements, or the possibility of measurement errors.

### Variable Selection Based on Significance and Change-In-Estimate

**Figure 1:**
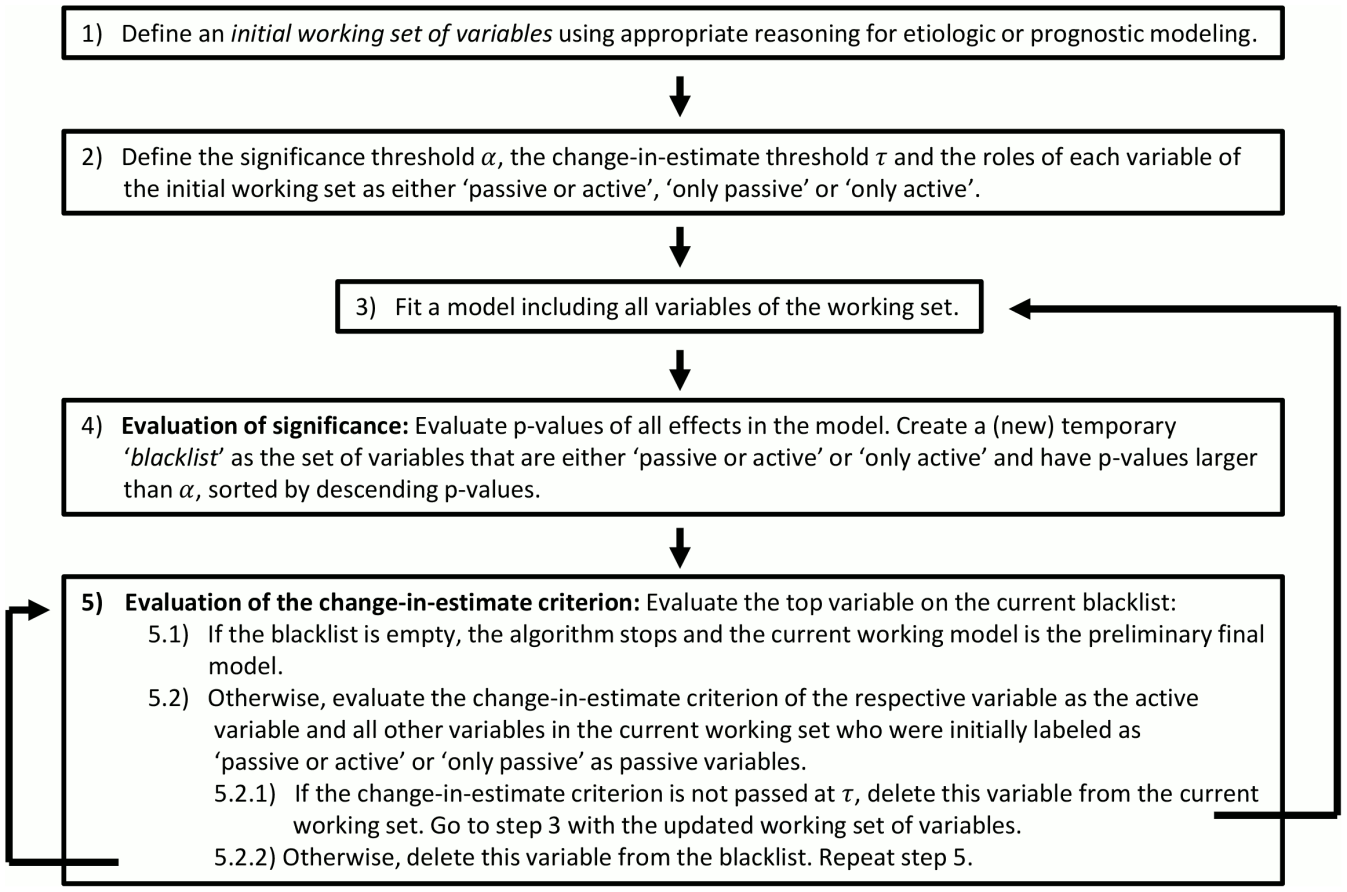
Brief outline of the augmented backward elimination procedure.

In summary, we propose to use BE augmented with a standardized change-in-estimate criterion on the quantity of interest for variable selection. We will denote this algorithm as ‘augmented backward elimination’ (ABE). The algorithm is briefly outlined in Figure 1. The ABE algorithm has been implemented in a SAS macro [13], which is described in more detail in a Technical Report [14]. The SAS macro can handle continuous, binary and time-to-event outcomes by implicitly applying linear regression using PROC REG, logistic regression using PROC LOGISTIC, or Cox proportional hazards regression using PROC PHREG, respectively. Basically, the ABE macro only needs the following specifications:

- Type of model (linear, logistic or Cox)
- Name of the outcome variable
- Names of the explanatory variables from the initial working set
- Roles of explanatory variables from the initial working set (‘passive or active’, ‘only passive’, ‘only active’)
- Significance threshold 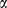 (default: 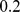)
- Change-in-estimate threshold 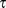 (default: 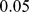)

Setting 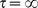 (i.e., to a very large number) turns off the change-in-estimate criterion, and the macro will only perform BE. On the other hand, the specification of 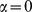 will include variables only because of the change-in-estimate criterion, as then variables are not safe from exclusion because of their p-values. Specifying 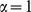 will always include all variables.

We agree with Hosmer and Lemeshow's position that any automated algorithm only suggests a *preliminary* final model. Such a model should be critically evaluated for possible extensions such as non-linear and non-additive (interaction) effects ([6], Chapter 5.2). Alternatively to the post-hoc inclusion of some transformations of continuous variables to allow for the estimation of non-linear effects, one could first apply an algorithm like ‘multivariable fractional polynomials' (MFP) which simultaneously selects variables and determines their functional form by appropriate transformations [5]. Then ABE could be applied by including the possibly transformed continuous variables and all other selected variables as ‘only passive’ variables, and any further variables which were not selected by MFP could be entered as ‘passive or active’ variables.

It should be mentioned that specifying a significance criterion of 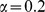 does not mean that the model itself or all its regression coefficients are significant at level 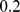. Simulations have shown that the actual significance levels of models derived by any variable selection procedure are usually much higher than the reported levels [25], [26]. Likewise, one should be aware that the actual confidence levels of the reported confidence intervals in the final model are often less than the nominal ones. Additionally, performance measures of the model such as 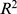 or area under the receiver operating characteristic curve are likely to be overestimated, i.e., too optimistic, if directly computed from the final estimates [27]. These phenomena are usually not dramatic if the sample size is large enough compared to the number of variables considered, e.g., if the effective sample size is at least 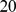 to 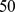 times the number of variables considered in the initial set. However, it can lead to wrong conclusions in other cases if not appropriately corrected [28].

Since the algorithm is available in a macro, it can easily be applied to bootstrap resamples or subsets of the data at hand, which allows to derive bootstrap confidence intervals for the regression coefficients (usually wider than their model-based counterparts), or to perform cross-validation to obtain optimism-corrected performance measures. In such analyses, the algorithm is applied to the resamples or subsets without any changes in the parameter settings. It may then result in different final models than obtained in the original analysis, and the final models may even differ between resamples or subsets. Thus, such analyses account for the variation in estimated regression coefficients that is produced by the uncertainty of variable selection in a data set, and they validate the *model development strategy* rather than the *model* itself. Later, we will demonstrate the difference between model-based and bootstrap standard errors by means of a real-life example.

### Simulation Study

We evaluated the proposed ABE procedure and compared it to BE, no selection and variable selection based on background knowledge in the setting of an etiologic study. Analyses comprised continuous, binary and time-to-event outcomes and were carried out using our SAS macro ABE.

We simulated seven normally distributed potential explanatory variables 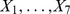 among which 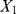 was the exposure variable of main interest. A latent outcome variable was defined as 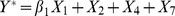. The covariance structure of 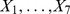 was defined such that omission of 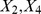 or 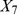, or false inclusion of 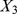 could induce bias into the estimate of 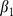, and that a pre-specified variance inflation factor (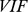) of 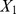 given 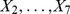 was attained. From 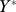 we generated continuous, binary and time-to-event variables to simulate linear, logistic and Cox regression, respectively. Further simulation parameters were set such that we obtained approximately equal sampling distributions of 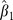 in these three types of regression analyses.

Specifically, 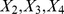
_,_ and 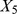 were drawn from a multivariate normal distribution with a mean vector of 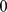, standard deviations of 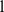 and bivariate correlation coefficients of 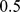
_._ 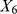 and 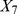 were independently drawn from a standard normal distribution. 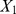 depended on 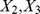 and 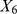 and was simulated from the equations 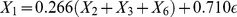 for scenarios with 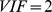 and 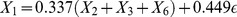 for 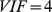, where 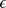 was a random number drawn from a standard normal distribution. The latent outcome variable was defined as 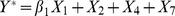.

Subsequently, we generated continuous, binary and time-to-event outcome variables 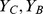 and 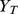 from 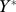 to simulate linear, logistic and Cox regression, respectively. In particular, 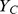 was drawn from a normal distribution with mean 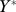 and standard deviation 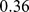. 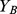 was drawn from a Bernoulli distribution with event probability 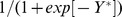. The overall expected event probability was approximately 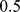. Weibull distributed survival times 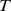 were drawn from 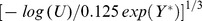, where 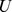 was a standard uniform random variable [29]. To obtain approximately 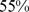 censoring (averaged over all scenarios), follow-up times 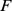 were drawn from a uniform 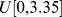 distribution, and the observable survival time and status indicators were defined as 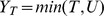 and 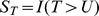, respectively. For Cox regression, all covariates were divided by 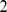. These definitions guaranteed that the sampling standard deviations of estimates of 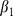 from linear, logistic and Cox regression in the scenarios with 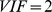 and 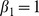 were approximately equal when the models were specified correctly.

In a factorial design we simulated 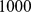 samples of 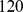 observations for each combination of true 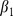 (either 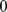 or 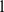), 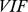 (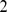,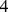) and type of regression (linear, logistic or Cox). If 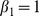 and 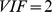, this sample size gave a power of 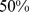 to reject the null hypothesis 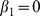 at a two-sided significance level of 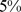 in all three types of regression, when the model was specified correctly. (In other words, in such models the expected p-value for this hypothesis was 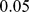.) Each sample was analyzed by a regression on all explanatory variables without selection, applying ABE with 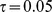 or 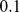 and 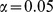 or 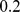, and applying BE with 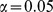 or 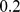. Unselected, ABE and BE analyses were then repeated by applying the disjunctive cause criterion [30] assuming that causal relationships between the variables 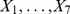 and their likely effects on the outcome were known, which means that 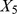 was eliminated from the scope of explanatory variables to consider.

For these evaluations, we used correctly specified models as ‘benchmark’, i.e., those containing 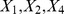 and 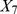 without further selection. We computed the bias and root mean squared error (RMSE) of unselected models, BE and ABE relative to the mean 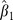 from such correctly specified models.

## Simulation Results

While the full results of our simulation study are contained in a Technical Report [14], the relative behavior of modeling by ABE, BE or by applying no variable selection can already be understood from the results selected for Table 1.

**Table 1:** Simulation study: bias and root mean squared error (RMSE) of regression coefficients 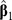 of a continuous exposure variable 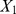 in unselected models, models selected by backward elimination (BE) and models selected by augmented backward elimination (ABE) for linear, logistic and Cox regression.

VIF	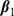	Variable selection among 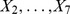	Bias (×100)	RMSE (×100)	Selected models (%)		
			of 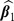		Biased	Correct	Inflated
Linear regression							
		No selection	1	24			100
2	0	BE, 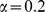	3	21	33	35	32
		ABE, 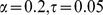 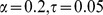	2	22	28	34	38
		No selection	1	24			100
2	1	BE, 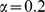	3	21	33	35	32
		ABE, 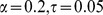	2	21	29	35	36
		No selection	1	57			100
4	0	BE, 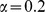	6	50	39	34	27
		ABE, 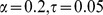	2	56	25	14	61
		No selection	1	57			100
4	1	BE, 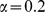	6	50	40	34	27
		ABE, 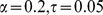	2	56	28	17	56
Logistic regression							
		No selection	1	20			100
2	0	BE, 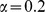	1	17	7	48	45
		ABE, 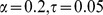	1	20	1	4	95
		No selection	6	25			100
2	1	BE, 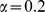	5	21	11	43	46
		ABE, 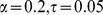	6	25	2	3	95
		No selection	2	47			100
4	0	BE, 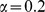 Geben Sie hier eine Formel ein.	4	38	12	46	42
		ABE, 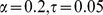	2	47	1	2	97
		No selection	6	55			100
4	1	BE, 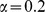	7	46	17	41	43
		ABE, 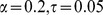	6	55	1	1	98
Cox regression							
		No selection	−1	22			100
2	0	BE, 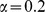 Geben Sie hier eine Formel ein.	1	19	20	40	40
		ABE, 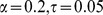	−1	22	7	13	80
		No selection	2	23			100
2	1	BE, 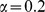 Geben Sie hier eine Formel ein.	3	20	19	40	41
		ABE, 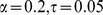	2	23	7	13	80
		No selection	−2	52			100
4	0	BE, 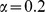	4	47	26	36	38
		ABE, 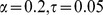	−2	52	6	5	89
		No selection	1	52			100
4	1	BE, 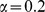 Geben Sie hier eine Formel ein.	6	46	28	36	36
		ABE, 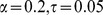	1	52	7	4	89

Abbreviations and symbols: 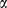, significance threshold; ABE, augmented backward elimination; BE, backward elimination; RMSE, root mean squared error; 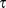, change-in-estimate threshold; VIF, variance inflation factor of 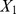 conditional on 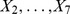. Sample size, 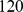 subjects; Number of simulations, 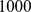; Variables selection based on six continuous candidate adjustment variables 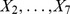, among which three are truly associated with the outcome and five are correlated with the exposure; ‘Biased’, at least one variable from the true model was not selected; ‘Correct’, selected set of variables matches the true model; ‘Inflated’, selected set of variables contains all variables of the true model and at least one further variable. Full details on the simulation setup are contained in the Methods section.

In general, we found that no selection and ABE selection lead to less biased estimates of the exposure effect than BE. The bias of ABE is small in absolute terms (usually around 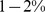 and only in logistic regression 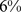) and never exceeding the bias of no selection. The bias of BE with 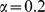, although slightly larger, is still acceptable for most practical purposes. BE with 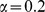 has some advantages with respect to RMSE compared to ABE and no selection. The RMSE of ABE is slightly smaller than that of no selection in linear regression, and both procedures yield virtually identical RMSEs in logistic and Cox regression. These observations can be explained by comparing the proportion of selecting ‘inflated’ and ‘biased’ models, i.e., models in which noise variables were falsely included or important variables were falsely excluded, respectively. Unselected models always contain such noise variables. In 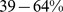 of the simulated data sets for linear regression, and in 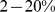 of the simulated data sets for logistic and Cox regression, ABE manages to identify and exclude those noise variables but occasionally also eliminates some of the important variables (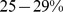 in linear regression, 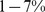 in logistic and Cox regression). By contrast, BE excludes noise variables more often (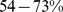), which likely explains its RMSE advantages. Note that despite BE's nominal significance level of 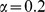, the probability of false inclusion of at least one noise variable lies in the range of 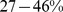 in our setting. Important variables are frequently missed by BE (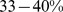 in linear regression, 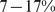 in logistic regression, 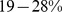 in Cox regression), and this causes a slightly higher bias.

In additional simulations which are only reported in the Technical Report [14], we found that lowering the significance level in BE to 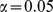 further increases BE's bias since important adjustment variables are more frequently missed, and this also causes a modest increase in RMSE. Furthermore, increasing the change-in-estimate threshold of ABE to 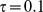 makes ABE more similar to BE, i.e., bias is increased but RMSE slightly decreased. With smaller samples, bias and RMSE are generally more inflated with all methods. Finally, incorporating background knowledge into variable selection improves bias and RMSE for all investigated selection procedures. Thus, we conclude that in the scenarios studied, application of ABE with the proposed settings for 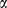 and 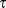 is at least as safe as application of BE with regard to bias, and is at least as good as, but often better than, including all available variables from the initial set for adjustment with regard to bias and RMSE.

## Example

Recently, Plischke et al. investigated the etiologic effect of urine osmolarity U_OSM_ (mosm/L) on progression to end stage renal disease defined as admission to dialysis in patients with chronic kidney disease [31]. The study was approved by the Medical University of Vienna's internal review board, No. 1982/2013. Of the 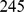 patients attending their nephrology outpatient clinic 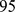 (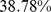) patients required dialysis during a median follow-up time of 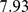 years. Here we want to elucidate the effect of different levels of U_OSM_, the exposure of interest, on the cause-specific hazard. Consequently, patients who died within follow-up but before initiating dialysis are considered as censored [32]. We used the logarithm to base 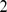 of U_OSM_ for all modeling because of its skewed distribution. Based on *a priori* knowledge nine explanatory variables measured at baseline are considered as potential confounders: log_2_ of creatinine clearance (ml/min), log_2_ of proteinuria (g/L), presence of polycystic kidney disease, whether or not beta-blockers, diuretics, or angiotensin-converting enzyme inhibitors and angiotensin II type 1 receptor blockers (ACEI/ARBs) were used, age in decades, gender, and mean arterial pressure (mmHg) (Table 2). We assume that all these variables fulfill the disjunctive cause criterion for selection of potential confounders, i.e., all variables are either a possible cause of the exposure or a possible cause of the outcome. The largest absolute correlation occurred between U_OSM_ and creatinine clearance (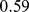), followed by three correlation coefficients slightly above 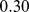 (use of diuretics and age; creatinine clearance and age; proteinuria and ACEI/ARBs).

**Table 2:** Urine osmolarity example: demographic and clinical characteristics of all 245 patients at baseline.

	Median (1^st^, 3^rd^ quartile) or Mean (SD) or n (%)
U_OSM_ (mosm/L)	510.1 (417.2, 620.6)
Creatinine clearance (ml/min)	46.4 (29.9, 78.8)
Proteinuria (g/L)	1.0 (0.4, 2.5)
Mean arterial pressure (mmHg)	97.7 (7.8)
Age (years)	54.6 (15.3)
Male gender	139 (56.7%)
Polycystic kidney disease	16 (6.5%)
Beta-blockers	116 (47.4%)
Diuretics	115 (46.9%)
ACEI/ARBs	206 (84.1%)
Log_2_ of U_OSM_	9.0 (0.5)
Log_2_ of creatinine clearance	5.6 (0.9)
Log_2_ of proteinuria	0.0 (1.6)

Depending on the scale of the characteristic and its distribution either the median (1^st^, 3^rd^ quartile), mean (standard deviation SD), or absolute number n (percentage) is given.

Abbreviations: ACEI/ARBs, use of angiotensin-converting enzyme inhibitors and Angiotensin II type 1 receptor blockers; SD, standard deviation; U_OSM_, urine osmolarity.

**Figure 2:**
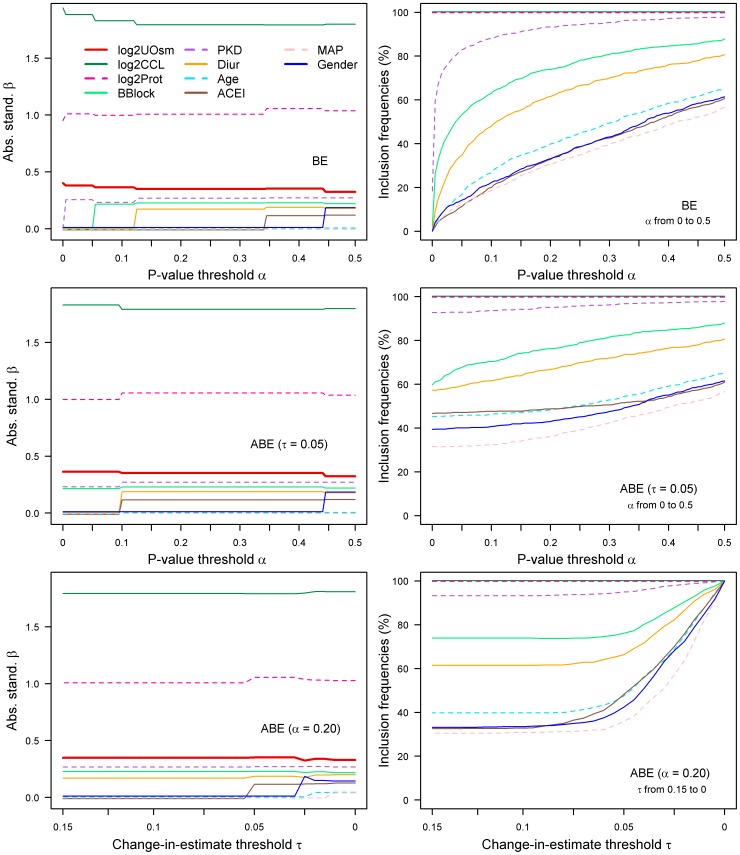
Urine osmolarity example: selection path (left column) of standardized regression coefficients 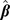 and model stability (inclusion frequencies) in 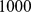 bootstrap resamples (right column) for backward elimination (BE) and augmented backward elimination (ABE). First row: BE with 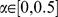; second row: ABE with 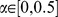 and 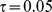
_;_ third row: ABE with 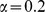 and 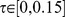. Abbreviations: ABE, augmented backward elimination; BE, backward elimination; log2UOsm, log2 of urine osmorality; log2CCL, log2 of creatinine clearance; log2Prot, log2 of proteinuria; BBlock, use of beta-blockers; PKD, presence of polycystic kidney disease; Diur, use of diuretics; Age, age in decades; ACEI, use of angiotensin-converting enzyme inhibitors and Angiotensin II type 1 receptor blockers; MAP, mean arterial pressure.

**Figure 3:**
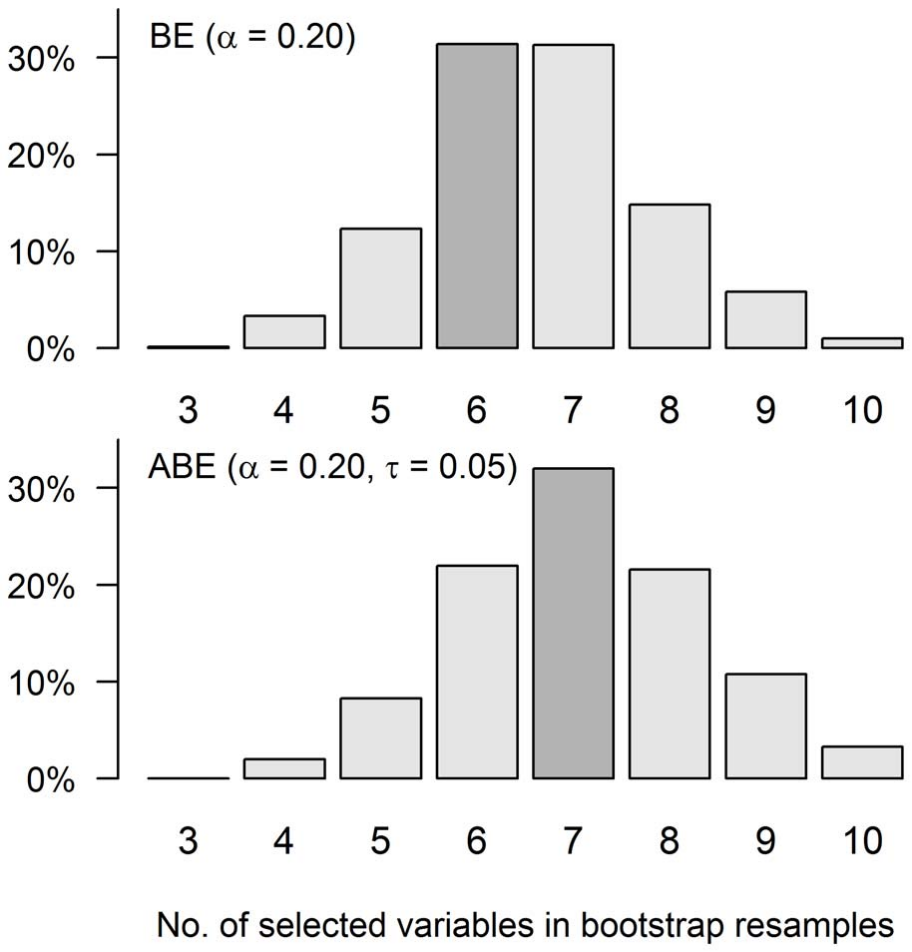
Urine osmolarity example: number of selected variables in the final models of 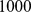 bootstrap resamples for backward elimination BE with 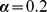 and augmented backward elimination ABE with 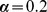 and 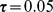. The highlighted bars indicate the number of selected variables in the original sample. Abbreviations and symbols: 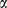, significance threshold; ABE, augmented backward elimination; BE, backward elimination; 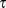, change-in-estimate threshold;.

The final model should be as simple as possible and should not include irrelevant variables. BE with a significance threshold 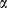 of 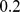 selects six of the ten variables from the initial set into the final model (Table 3). Figure 2 (first row, left column) shows the sensitivity of the absolute standardized regression coefficient of U_OSM_ on the choices of 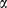. Model stability was assessed by inclusion frequencies of each variable in 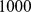 bootstrap resamples, each analyzed with BE and 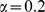. All explanatory variables selected into the (original) final model by BE are selected in at least 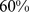 of all bootstrap resamples. Figure 3 (first row) shows the number of selected variables in the 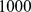 models of the bootstrap resamples. In 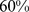 of the bootstrap resamples six to seven variables were selected. The sensitivity of the bootstrap inclusion frequencies on the choice of significance threshold 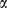 is shown in Figure 3 (first row, right column).

**Table 3:** Urine osmolarity example: final models selected by backward elimination (BE) with a significance threshold 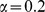, augmented backward elimination (ABE) with 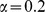 and a change-in-estimate threshold 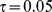, and unselected model (No selection).

Parameter	BE ( 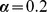 ) HR (95% CI), p	Bootstrap inclusion frequencies	ABE ( 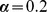 , 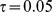 ) HR (95% CI), p	Bootstrap inclusion frequencies	No selection HR (95% CI), p
Log_2_ of U_OSM_	2.03 (1.11, 3.71), 0.021		2.05 (1.13, 3.72), 0.019		1.95 (1.03, 3.72), 0.042
Log_2_ of creatinine clearence	0.14 (0.09, 0.21), <0.001	100.0%	0.14 (0.09, 0.21), <0.001	100.0%	0.13 (0.08, 0.21), <0.001
Log_2_ of proteinuria	1.88 (1.61, 2.19), <0.001	100.0%	1.94 (1.64, 2.29), <0.001	100.0%	1.90 (1.60, 2.25), <0.001
Polycystic kidney disease	2.94 (1.50, 5.80), 0.002	93.1%	2.98 (1.51, 5.88), 0.002	94.3%	2.95 (1.48, 5.87), 0.002
Beta-blockers	1.57 (1.02, 2.44), 0.042	74.2%	1.58 (1.02, 2.446), 0.040	77.1%	1.55 (0.99, 2.42), 0.057
Diuretics	1.41 (0.91, 2.16), 0.122	60.6%	1.45 (0.94, 2.24), 0.096	66.3%	1.49 (0.94, 2.38), 0.092
ACEI/ARBs		35.2%	0.71 (0.35, 1.45), 0.344	47.7%	0.69 (0.33, 1.42), 0.310
Age (in decades)		36.9%		45.9%	0.96 (0.83, 1.11), 0.593
Male gender		33.0%		40.4%	1.14 (0.72, 1.83), 0.577
Mean arterial pressure		30.1%		36.8%	1.01 (0.97, 1.04), 0.730

Urine osmolarity U_OSM_, the exposure of main interest, is included in all models. The initial set of adjustment variables for these models was selected by the disjunctive cause criterion. Hazard ratios (HR), 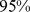 confidence limits (CI) and p-values are given. Model stability was evaluated by bootstrap inclusion frequencies (based on 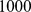 bootstrap resamples). U_OSM_, creatinine clearance, and proteinuria were log_2_-transformed and therefore, corresponding hazard ratios are per doubling of each variable.

Abbreviations and symbols: 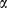, significance threshold; ABE, augmented backward elimination; ACEI/ARBs, use of angiotensin-converting enzyme inhibitors and Angiotensin II type 1 receptor blockers; BE, backward elimination; CI, confidence interval; HR, hazard ratio; 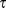, change-in-estimate threshold; U_osm_, urine osmolarity (mosm/L).

Applying ABE with 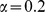 and 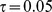 additionally selects ACEI/ARB use, since this causes a change in the standardized hazard ratio of proteinuria by more than 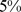. ACEI/ARB use is included in almost 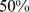 of all bootstrap resamples. Figure 3 (second row) also shows that ABE tends to select slightly more variables than BE. From a medical point of view the inclusion of ACEI/ARBs into the model can be explained, as ACEI/ARBs inhibit the activity of the renin-angiotensin-aldosterone system (RAAS), which controls fluid and electrolyte balance through effects on the heart, blood vessels and the kidneys, and have been shown to be renoprotective and slow the progression of chronic nephropathies [33]. Angiotensin II, the main effector of the RAAS, exerts a vasoconstrictory effect on postglomerular arterioles, increasing glomerular hydraulic pressure and ultrafiltration of plasma proteins. Additionally, Angiotensin II has been linked to sustained cell growth, inflammation and fibrosis, which have also been associated with accelerated renal damage.

Confidence limits and p-values given in Table 3 do not reflect model uncertainty and hence, are likely to underestimate the variability of regression coefficients. Table 4 shows bootstrap standard errors for U_OSM_ which are clearly higher than their model-based counterparts. Robust standard errors correct some but not all of the uncertainty induced by model selection and may be a good compromise if full resampling cannot be applied [34].

**Table 4:** Urine osmolarity example: incorporating model uncertainty into standard error (SE) estimates of urine osmolarity U_OSM_.

Selection algorithm	Model-based SE	Robust SE	Bootstrap SE
BE with 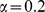	0.307	0.346	0.400
ABE with 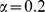 , 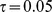	0.305	0.340	0.400

Model-based standard error, robust standard error and standard error based on 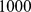 bootstrap resamples for models selected with backward elimination (BE) and augmented backward elimination (ABE).

Abbreviations and symbols: 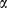, significance threshold; ABE, augmented backward elimination; BE, backward elimination; 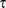, change-in-estimate threshold; SE, standard error; U_osm_, urine osmolarity (mosm/L).

Up to now, all variables from the initial set were used as ‘passive or active’ variables when evaluating the change-in-estimate criterion. If required, we could define only U_OSM_ as ‘passive or active’ and treat all other explanatory variables as ‘only active’. Then only explanatory variables which reach the significance threshold 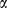 or change the standardized hazard ratio of U_OSM_ by more than 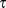 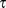 will be selected into the final model. Applying ABE with such redefined roles of variables and with 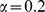 and 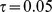 gives the same final model as selected by BE with 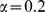.

## Discussion

In biomedical research we are often confronted with complex statistical modeling problems involving a large number of potential explanatory variables and only restricted prior knowledge about their relationships. Therefore, practical and reproducible approaches to statistical modeling are needed.

The first step in finding a practically useful statistical model should always be a careful pre-selection of explanatory variables based on subject-matter knowledge. Often this is the most important prerequisite for any analytical modeling steps to follow. If enough subject-matter knowledge is available causal diagrams may be of help. However, causal diagrams are always based on expert knowledge and opinions and their construction may sometimes not be universally reproducible. This may motivate the careful use of a reproducible data-driven variable selection procedure.

Based on our evaluation of unselected models, ABE and BE, we recommend ABE for development of statistical models when there is only little guidance on which variables to include. Compared to BE, ABE more often avoids bias due to the false exclusion of an important confounding variable. Compared to no variable selection, ABE frequently supplies smaller and thus practically more useful models but with no detrimental consequences on bias or RMSE. By construction, ABE models essentially show only negligible differences compared to unselected models including all candidate variables. In practice, this may be important to demonstrate to reviewers and readers of a research report that all relevant confounders are accounted for.

Our proposal for standardization of the change-in-estimate criterion employed by ABE focuses on the quantity of interest in a given type of regression analysis (regression coefficients, hazard ratios or odds ratios). It also considers the scaling of the variables, such that its results are invariant to linear transformations of variables. ABE can be adopted to the statistical modeling problem at hand, by defining the role and thus the importance of each candidate explanatory variable. Our approximation of the change-in-estimate shows that a ‘significant’ change-in-estimate always results if the variable in question has a significant effect on the outcome. Thus, if ‘false positive’ selections are to be avoided, a simple significance-based selection such as BE is the method of choice. However, even though ABE and other data-driven variable selection methods may be useful statistical tools, they should not be a replacement for careful thinking of possible causal relationships.

Whenever data-dependent variable selection is conducted, reported standard errors and confidence limits understate the true uncertainty of regression coefficients and derived quantities (hazard or odds ratios). We have demonstrated how to use resampling-based methods to obtain more reliable interval estimates and to evaluate model stability.

We have written a SAS macro ABE, which implements augmented backward elimination for linear, logistic and Cox regression. By means of a simulation and an analysis of a biomedical study, we evaluated the ABE algorithm and its implementation in SAS. Depending on the settings of the parameters of ABE (significance threshold 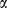, change-in-estimate threshold 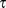 and roles of candidate explanatory variables), the number of variables in the final model selected by ABE will be between the number of variables selected by BE and the total number of variables. Based on our simulations and practical experiences with ABE, we suggest to use a significance threshold of 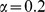 and a change-in-estimate threshold of 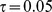. The SAS macro ABE is freely available under a General Public License (GPL) at: http://cemsiis.meduniwien.ac.at/en/kb/science-research/software/statistical-software/abe/.

## Supporting Information

Materials S1 SAS code to reproduce the simulation study and the analysis of the urine osmolarity example.
(ZIP)
