# Supplementary material for: Augmented Backward Elimination: A Pragmatic and Purposeful Way to Develop Statistical Models
Source: PLoS One. 2014 Nov 21;9(11):e113677. doi: 10.1371/journal.pone.0113677 (PMC4240713; doi:10.1371/journal.pone.0113677)
Supplement: Materials S1 — SAS code to reproduce the simulation study and the analysis of the urine osmolarity example. (ZIP) [file pone.0113677.s001.zip › Materials S1.pdf]

Materials S1

Dunkler D, Plischke M, Leffondré K, Heinze G (2014):

Augmented Backward Elimination: a pragmatic and purposeful way to develop statistical models.

Files included in the Supporting_Information zip-file:

- ABE.sas: `%ABE` SAS macro (Version 2014.05)
- ABE_Bootstrap.sas: `%ABE_Bootstrap` SAS macro (Version 2014.05)

- Files for simulation study:
  - ABE_simulation.sas: Runs the simulation study for linear, logistic and Cox regression as described in the paper.
  - Macros_for_simulation.sas: further SAS macros used by ABE_simulation.sas.

- Analysis of urine osmolarity study:
  - urine osmolarity data.xls: data set used in the paper
  - example.sas: Runs the example analysis of the urine osmolarity study as described in the paper.

The most recent version of the `%ABE` SAS macro and a Technical Report describing its use is freely available under a General Public License (GPL) at http://cemsiis.meduniwien.ac.at/en/kb/science-research/software/statistical-software/abe/ .
